# Purification and Characterization of an ATPase GsiA from* Salmonella enterica*

**DOI:** 10.1155/2017/3076091

**Published:** 2017-06-12

**Authors:** Zhongshan Wang, Meng Zhang, Xiaodong Shi, Quanju Xiang

**Affiliations:** ^1^Jiangsu Province Key Laboratory of Anesthesiology, Xuzhou Medical University, Xuzhou, China; ^2^Department of Gynecology, Central Hospital of Xuzhou, Affiliated Hospital of Southeast University, Xuzhou, China; ^3^Department of Microbiology, College of Resource and Environmental Sciences, Sichuan Agricultural University, Chengdu, China

## Abstract

The coding sequence of* Salmonella enterica gsiA* was cloned and expressed in* E. coli*. The protein was purified and ATPase activity was characterized by NADH oxidation method. GsiA exhibited optimum activity at 30°C and at pH 8 in Tris/HCl buffer. GsiA protein was stable at 20°C. 66% and 44% activity remained after incubation at 30°C and 40°C for 30 min. pH 7 and pH 9 incubation would obviously reduce the ATPase activity. In vivo functionality of* gsiA* was determined by constructing gene deletion strains.* gsiA* was shown to be essential for GSI mediated glutathione uptake and* gsiA* deletion could decrease the virulence of* Salmonella enterica*. Interactions of glutathione import proteins GsiA, GsiB, GsiC, and GsiD were investigated by using bacterial two-hybrid system. GsiA could interact with itself and inner membrane proteins GsiC and GsiD. This report provides the first description of* gsiA* functions in* Salmonella enterica*. The results could help elucidating the glutathione uptake mechanism and glutathione functions in bacteria.

## 1. Introduction

Glutathione (*γ*-L-glutamyl-L-cysteinyl-glycine; GSH) is a vital intracellular cysteine containing antioxidant across all kingdoms of life. Glutathione usually attains mM concentrations in cells and assumes a plethora of cellular roles, such as control intracellular redox homeostasis, and protects against oxidative and xenobiotic stresses by glutathionylation [1, 2]. Glutathione also functions in cell signaling [3] and salvage of cysteine [4]. Glutathione manifests inside the cell predominantly in the thiol-reduced form (GSH, >98%) [5]. The remaining amounts of glutathione are in the oxidation form: glutathione disulfide (GSSG) or disulfides with target proteins [6]. Compounding evidence indicates that glutathione import by yeasts and bacteria could serve as a supply of organic sulfur [7–9]. However, physiological importance of glutathione import still needs further investigation.

H^+^ symporter [10], Na^+^-dependent transporter [11], and glutathione S conjugate exporters [12] play important roles in transport glutathione and its derivatives in and out of cells. However, the first bacterial glutathione importer (GSI) with an ATP binding cassette was identified in 2005 [7]. This importer consists of* gsiA*,* gsiB*,* gsiC*, and* gsiD*, encoding the ATP binding protein, the periplasmic glutathione binding protein, and two plasma membrane components, respectively [13].

Usually, the ATP binding components could hydrolyze ATP, providing energy for substrate transport [14]. Glutathione import in* Escherichia coli* is energy dependent which is supported by GsiA. The ATP binding subunits of transporters usually worked as a dimer [15]. The nucleotide binding pocket will open and close for ATP or ADP binding, which make the protein intimately related to translocation [16] by regulating ATPase activity. Whether GsiA serves as a dimer and how it regulates the glutathione import are still unclear.

Herein, the spectrum of expression, purification, and characterization of* gsiA* from* Salmonella enterica*, a bacterial pathogen responsible for enteritis and typhoid fever, was described. MBP (maltose binding protein) was employed as a fusion protein. The in vivo and in vitro studies of* gisA* were carried out. The substrate specificity of GsiA and its stability as function of temperature and pH were investigated. Studies of GsiA will help to clarify the mechanism and functions of glutathione uptake in bacteria.

## 2. Materials and Methods

### 2.1. Strain and Chemicals

Expression vectors plou3 and pBAD24 were kind gifts of Professor Changjiang Dong from University of East Anglia. Plasmids pET11a-link-NGFP, pMRBAD-link-CGFP, pN-Z, and pC-Z were kindly gifted by Professor Lynne Regan of Yale University. The* Salmonella enterica *subsp.* enterica *serovar Typhimurium* LT2: STM0848* and* E. coli* strains BL21(DE3) and DH5*α* were preserved in our laboratory. KOD-FX polymerase was bought from ToYoBo, and T4 DNA ligase and restriction enzymes were got from NEB. Other chemicals were purchased from Sigma-Aldrich.

### 2.2. Heterologous Expression of gisA

*gsiA* was amplified from genomic DNA of* Salmonella enterica* by standard using primers gsiA-F and gsiA-R (Table 1) and cloned into plou3. The recombinant plasmid, referred to as plou3-gsiA, was transformed into BL21(DE3) for expression.

The strain was grown in Luria-Bertani (LB) medium containing ampicillin (100 *μ*g/mL) at 37°C. Protein expression was induced by addition of 0.1 mM IPTG at an OD_600_ of 0.5–0.6 and grown at 20°C for 20 h. Cells were harvested by centrifugation at 5000*g* for 15 min at 4°C.

### 2.3. Purification of GsiA Recombinant Protein in* E. coli*

The cell pellet was resuspended in 50 mM Tris/HCl pH 8, 100 mM NaCl, 15 mM imidazole, 1 mM phenylmethanesulfonyl fluoride (PMSF), 1 *μ*M lysozyme, 1 *μ*M DNase, and a protease inhibitor cocktail (Sigma, one tablet per 100 mL). The cell was disrupted by homogenizer FB-110X (LiTu, China) at 1000 MPa. The cell debris was removed by centrifugation at 10,000*g* for 15 min at 4°C. The supernatant was loaded onto Ni^2+^-NTA affinity column (GE Healthcare) and washed with 30 mM imidazole, 50 mM Tris/HCl pH 8, and 300 mM NaCl. GsiA-MBP was eluted with 300 mM imidazole, 50 mM Tris/HCl pH 8, and 300 mM NaCl. The imidazole was removed by using desalting column (GE Healthcare, buffer containing 50 mM Tris/HCl pH 8, 300 mM NaCl). The protein was digested with TEV protease and purified by MBP column. The flow through GsiA protein was further purified by a size exclusion column Superdex 200 (GE Healthcare) with 50 mM Tris/HCl pH 8, 300 mM NaCl, and 5% (v/v) glycerol. The purity of GsiA was analyzed on 12% (v/v) SDS-PAGE. GsiA protein conformations were analyzed on 12% (v/v) native gel. The pure GsiA protein was concentrated (30 kDa molecular weight cutoff tube) (Sartorius) to about 10 mg/mL, which was measured by Nanodrop 2000 (Thermo Scientific).

### 2.4. Immunoblot Analysis

Immunoblot analysis to confirm the GsiA expression was performed as previously described [17], with anti-His monoclonal antibody (Abcam, anti-His, 400 *μ*g/mL, 1 : 1000 (v/v) dilution) and horseradish peroxidase labeled antibody (Abcam, goat anti-mouse, 0.8 mg/mL, 1 : 5000 (v/v) dilution).

### 2.5. GsiA Interacts with Other Components

The interaction of GsiA with other components of glutathione importer was analyzed by using GFP fragments reassembly protocol [18, 19].* gsiA*,* gsiC*, and* gsiD* genes were cloned into pET11a-link-NGFP.* gsiA*,* gsiB*,* gsiC*, and* gsiD* genes were cloned into pMRBAD-link-CGFP. Recombinant plasmids carrying N- and C-fragment of GFP were simultaneously transformed into BL21(DE3) using 10 ng of each construct. The cells were plated on LB plates containing kanamycin (35 *μ*g/mL) and ampicillin (100 *μ*g/mL). Single colony harboring two plasmids was obtained and incubated overnight with shaking at 37°C. Fresh overnight culture was diluted (1 : 100) and 10 *μ*L medium was plated onto screening media, containing 10 *μ*M IPTG and 0.2% arabinose. The plates were incubated at 20°C for 2 days, which will give reproducible green-fluorescent colonies.

### 2.6. Enzymatic Characterization of GsiA

The ATPase activity of GsiA was determined by measuring NADH oxidation [17, 20] through recording the decrease of absorbance at 465 nm (*λ*_ex_ = 340 nm, *λ*_em_ = 465 nm) with a Nanodrop 2000 (Thermo Scientific).

### 2.7. *gsiA* In Vivo Function Assay

Glutathione is supposed to be imported into Gram-negative bacteria mainly through *γ*-glutamyltranspeptidase (GGT) or GSI method [7]. To characterize function of GisA, the* gsiA* and* ggt* gene deletion mutant of* Salmonella enterica* was constructed with *λ*Red recombination system [21, 22]. The kanamycin fragment in pKD4 was amplified by using primers gsiA del-F and gsiA del-R (Table 1). The PCR product with 58 bp upstream and 58 bp downstream homologous to adjacent regions of* gsiA* was digested with* Dpn*I and gel-purified. pKD46 was transformed into* Salmonella enterica* by traditional CaCl_2_ method. The cell was grown in SOB medium and grown at 30°C to an OD_600_ of 0.5–0.6. 2 mM L-arabinose was added 1 h before cell collection. Competent cells were obtained by washing the pellet with ice-cold 10% glycerol. The cells were subjected to electroporation by MicroPulser (Bio-Rad) using a 0.1 cm chamber with 50 *μ*L of the competent cell and 50 ng of PCR product.

*gsiA* gene deletion was verified by PCR with primers gsiA-F1 and gsiA-R1. The* gsiA* deletion strain was made competent and* ggt* gene was deleted as above. The chloramphenicol fragment in pKD3 was amplified with primers ggt del-F and ggt del-R (Table 1), generating a product with 56 bp upstream and 58 bp downstream homologous to adjacent regions of* ggt*.

The cell growth and glutathione uptake curves of* gsiA* deleted* Salmonella enterica *strains were measured. M9 medium [23] was used as minimal medium with MgSO_4_ replaced by MgCl_2_. Reduced glutathione (1 mM, ≥98%) was served as the only sulfur source. Plasmid pBAD24-gsiA was constructed (pBAD24 plasmid and* gsiA* primers F1 and R1 were used) and transformed into* gsiA* deleted strain to compensate this defection.

6- to 8-week-old male Kunming mice were employed in present study, which were provided by Experimental Animal Center of Xuzhou Medical University. The mice were infected intraperitoneally with wild type and* gsiA* deletion strain for 10^4^ bacteria per mouse. The mice were sacrificed at 24 h and 72 h after infection. The livers and spleens were taken out and CFU counts of viable bacteria were recorded [24]. The experimental protocol according to the Declaration of National Institutes of Health* Guide for Care and Use of Laboratory Animals* (Publication Number 80–23, revised 1996) was approved by the Animal Care and Use Committee of Xuzhou Medical University (Xuzhou, China).

## 3. Results and Discussion

### 3.1. Expression and Purification of GsiA

The coding sequence of the* gsiA* gene was amplified from* Salmonella enterica *subsp.* enterica *serovar Typhimurium* LT2: STM0848* genome and cloned into pMAL-c2X derived plou3 vector. To facilitate protein purification, a 6×His tag was added before MBP and a TEV protease cleavage site was added between MBP and polylinker. The resultant plasmid was denominated by plou3-*gsiA*. The insertion of* gsiA* was confirmed by DNA sequencing.

**Figure 1:**
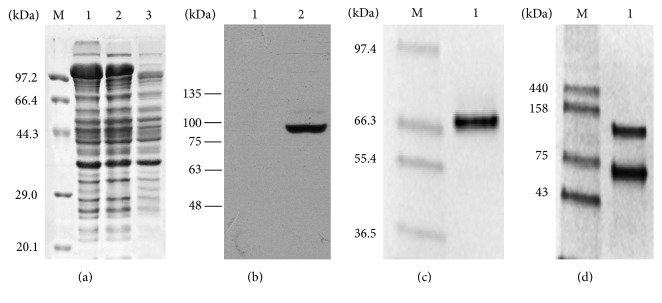
Overexpression and purification of GsiA. (a) SDS-PAGE analysis of GsiA expression. M: marker; lanes 1-2: total protein and soluble fraction of GsiA in BL21(DE3) induced with 0.1 mM IPTG at 20°C for 20 h; lane 3: total protein without IPTG induction. (b) Western blot confirmation of GsiA-MBP expression. Immunoblot of the gel decorated with anti-His tag antibody. Lanes 1-2: total protein of GsiA in BL21(DE3) grown at 20°C for 20 h induced with no IPTG and 0.1 mM IPTG. (c) The GisA protein was separated on 12% (v/v) SDS-PAGE and purity was analyzed with QuantiyOne software. M: marker; lane 1: purified GsiA protein. (d) Protein conformations in Tris buffer (containing 50 mM Tris/HCl pH 8, 300 mM NaCl, and 5% (v/v) glycerol) were confirmed by 12% (v/v) native gel. M: marker (GE Healthcare); lane 1: purified GsiA protein.

The expression conditions of GsiA-MBP in BL21(DE3) were optimized. 0.1 mM IPTG induced at 20°C for 20 h will give high productivity of soluble GsiA (Figure 1(a)). SDS-PAGE analysis showed that the molecular mass of GsiA was about 70 kDa, in accordance with prediction (Figure 1(c)).

GsiA-MBP fusion protein was firstly purified by Ni^2+^ column. Incubation of GsiA-MBP with TEV protease could separate GsiA from MBP. After MBP and gel filtration column purification, the purity level of GsiA was in excess of 95% (Figure 1(c)). The purified GsiA was shown to have two conformations in gel filtration buffer (Figure 1(d)). The pure protein was concentrated to 12 mg/mL and used for ATPase activity assay. Approximately 1.6 mg of GsiA protein was obtained from 1 L bacteria culture.

Western blot was carried out to confirm the expression of GsiA. As there was a 6×His tag at the N terminal of the fusion protein, anti-His antibody could be used to detect the expression of GsiA-MBP (Figure 1(b)).

### 3.2. Characterization of ATPase Activity of GsiA

**Figure 2:**
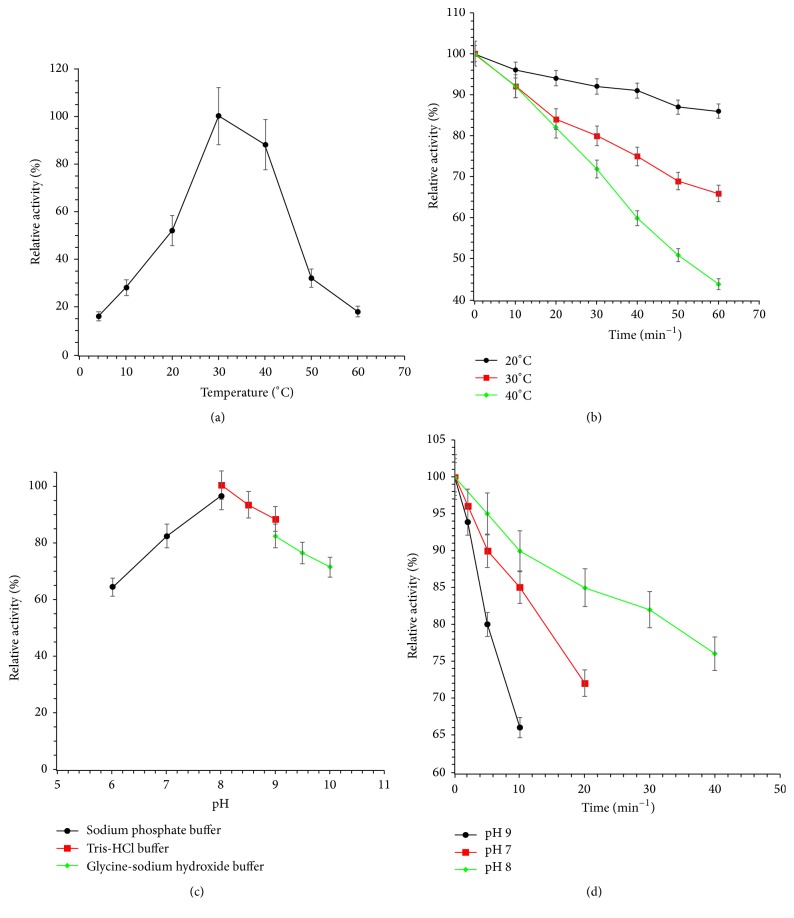
Characterization the ATPase of GsiA protein. (a) Effect of temperature on GsiA activity determined in Tris/HCl buffer (pH 8) at 4–60°C. (b) Thermostability assay. The purified GsiA protein was incubated in Tris/HCl buffer (pH 8) at 20, 30, and 40°C. Aliquots were collected at specific time points for residual activity assay at 30°C in Tris/HCl buffer (pH 8). (c) Effect of pH on GsiA protein activity. The enzyme activity was measured at 30°C in different buffers with pH varied from 6 to 10. (d) pH stability assay. The GsiA was incubated at pH 7, 8, or 9; aliquots were collected at different time points for residual activity assay. The error bars represent the mean ± standard deviation (*n* = 3).

**Figure 3:**
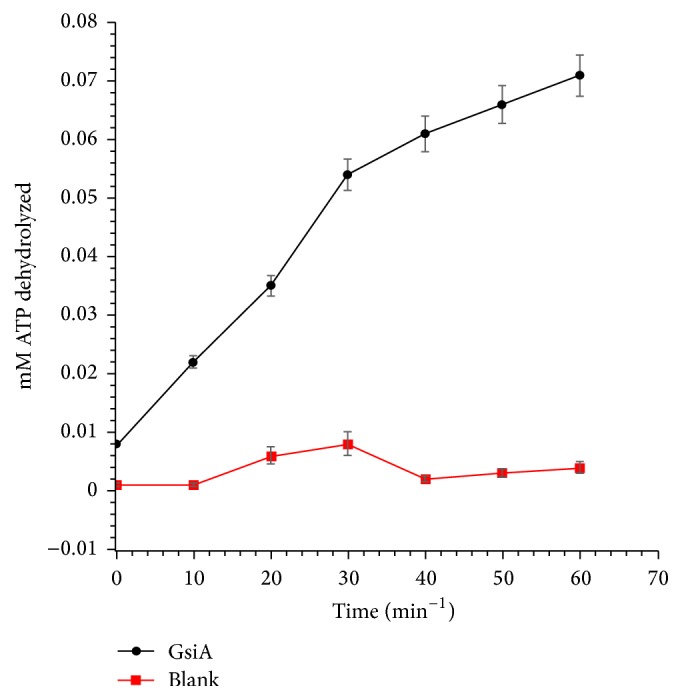
The ATPase activity of GsiA was determined by measuring NADH oxidation through recording the decrease of absorbance at 465 nm. The reaction was carried out in 50 mM Tris/HCl pH 8, 300 mM NaCl, and 5% (v/v) glycerol, containing 0.8 *μ*M GsiA, 5 mM MgCl_2_, BSA (0.1 mg/mL), lactate dehydrogenase (0.1 units/mL), 4 mM phospho(enol) pyruvic acid, pyruvate kinase (6 units/mL), and 0.32 mM *β*-nicotinamide adenine dinucleotide, reduced dipotassium (NADH). The reaction was initiated by adding 1 mM ATP. Reaction buffer without GsiA protein was recorded as control (Blank). Known concentrations of NADH in reaction buffer were used to generate the standard curve. The error bars represent the mean ± SD (*n* = 3).

The purified GsiA exhibited optimum activity at 30°C and at pH 8 in Tris/HCl buffer (Figure 2). The GsiA protein was stable at 20°C. 66% and 44% activity remained after incubation at 30°C and 40°C for 30 min, respectively (Figure 2(b)). The protein was pH sensitive. pH 7 and pH 9 incubation would obviously reduce the ATPase activity (Figure 2(d)). One enzyme unit was defined as the amount of protein converting 1 *μ*M ATP to ADP per min at pH 8 and 30°C. 1 *μ*M GsiA could transform 78.8 *μ*M ATP into ADP in 1 h (Figure 3).

### 3.3. Protein Interaction of GsiA with Other Components

**Figure 4:**
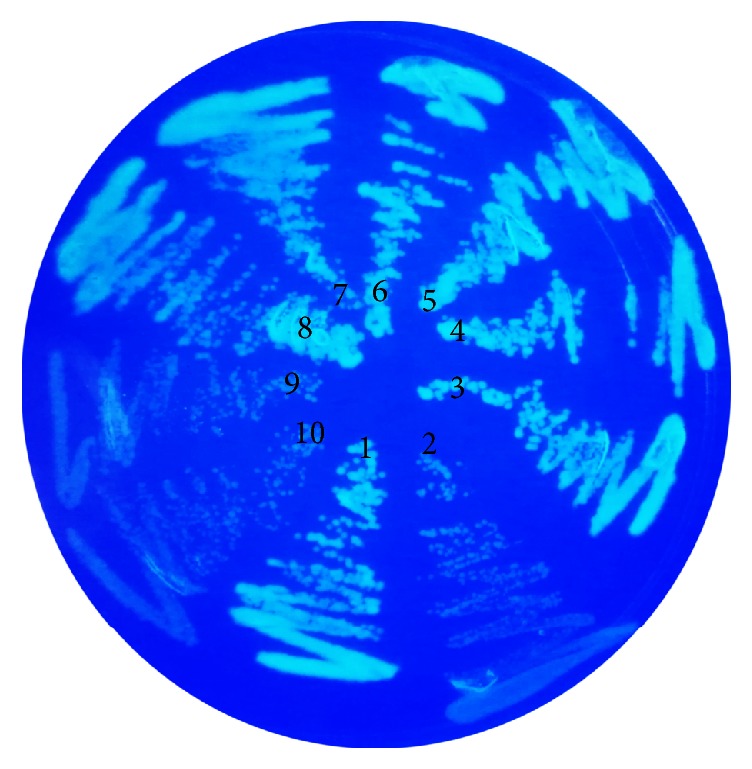
In vivo analysis of GsiA interaction with other components of glutathione importer. The genes were cloned into pET11a-link-NGFP and pMRBAD-link-CGFP which were referred to as pN and pC. pN-Z and pC-Z were positive control plasmids. BL21(DE3) transformants were induced with 10 *μ*M IPTG and 0.2% arabinose. The plates were incubated at 20°C for 2 days. If proteins could interact with each other, the cell showed reproducible green fluorescence under UV light. Numbers 1 to 10 were cells transformed with pN-gsiA and pC-gsiA; pN-gsiA and pC-gsiB; pN-gsiA and pC-gsiC; pN-gsiA and pC-gsiD; pN-gsiC and pC-gsiD; pN-gsiC and pC-gsiA; pN-gsiD and pC-gsiA; pN-Z and pC-Z; pN-gsiA and pC-Z; pN-Z.

To determine the interaction of GsiA with other proteins of glutathione import system,* gsiA*,* gsiC*, and* gsiD* in pET11a-link-NGFP and* gsiA*,* gsiB*,* gsiC*, and* gsiD* in pMRBAD-link-CGFP were pairwise simultaneously transformed into BL21(DE3). 10 *μ*M IPTG and 0.2% arabinose were used to induce the protein expression, which make GFP reassembly possible. The assembled GFP usually showed fluorescence in vivo (Figure 4), especially when activated by UV light. Protein interaction assay showed that GsiA could interact with the trans-membrane proteins GsiC and GsiD (Figure 4). The most interesting phenomenon was that GsiA showed interaction with itself. Native gel showed that GsiAcould oligomerize in gel filtration buffer. This might be explained that GsiA worked as a dimer (Figure 1(d)), like most of ATP binding proteins in ABC cassette superfamily. GsiA did not interact with GsiB. It might be because of their different cell location. GsiA and GsiB were predicted to be located in the periplasm and cytoplasm of cell, respectively [13].

### 3.4. *gsiA* gene Was Essential for GSI Mediated Glutathione Import

**Figure 5:**
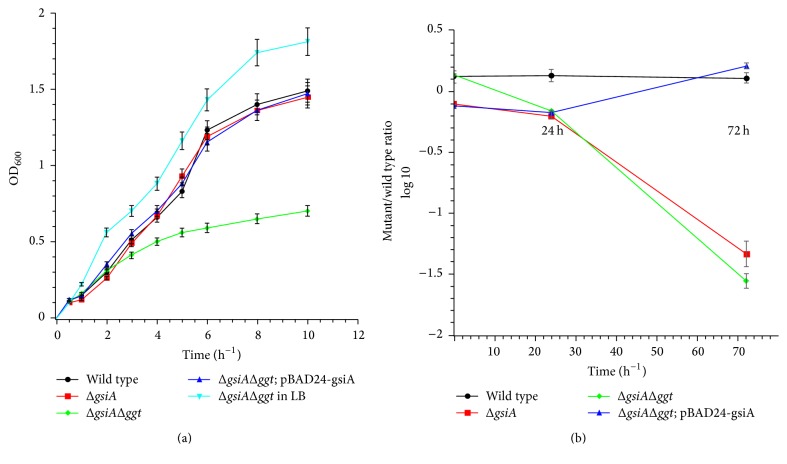
Effects of GsiA on cell growth and strain virulence. (a)* gsiA* and* ggt* gene deletion strain were constructed. The growth curves of mutant and wild type* Salmonella enterica* were analyzed. pBAD24-gsiA was transformed into the* ΔgsiAΔggt* to compensate the defection. (b) The virulence of* Salmonella enterica* was determined by recording the CFU counts of viable bacteria from livers and spleens.

**Figure 6:**
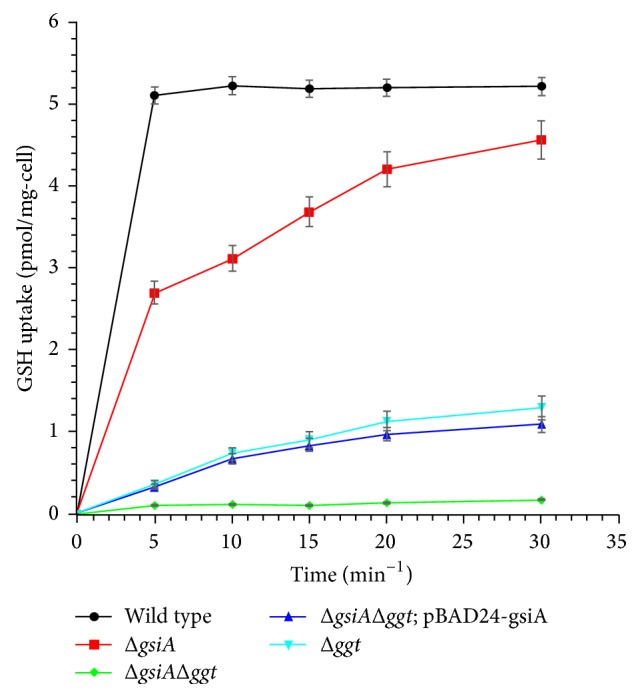
Effects of GsiA on glutathione uptake.* gsiA* and* ggt* gene deletion strains were constructed. The glutathione uptake curves of mutant and wild type* Salmonella enterica* were analyzed. M9 medium was used as minimal medium with MgSO_4_ replaced by MgCl_2_. Reduced glutathione (1 mM, ≥98%) was served as the only sulfur source. Glutathione concentration in the medium was measured by Glutathione Assay Kit (Sigma) according to the instruction manual.

The* gsiA* and* ggt* gene were replaced by kanamycin and chloramphenicol resistant gene, respectively. The resulting* Salmonella enterica* strain was named* ΔgsiA* and* ΔgsiAΔggt*. The gene deletion was verified by PCR (data not shown). M9 medium was used to cultivate the mutant with glutathione as the sole sulfur source. The cell growth and glutathione uptake curves of wild type and mutant strains were measured. Compared with cultivation in LB broth, wild type strain grown in glutathione containing M9 medium was not affected, showing that glutathione could serve as sulfur source (Figure 5(a)). The growth of* ΔgsiA* was also not affected. However,* ΔgsiAΔggt* strain which grew in glutathione containing M9 medium was slower than in LB medium. While pBAD24 empty vector had no effect on cell growth, pBAD24-gsiA transformed* ΔgsiAΔggt* strain could completely compensate this defection (Figure 5(a)). The glutathione uptake in* ΔgsiAΔggt* strain was undetectable (Figure 6).* gsiA* gene deletion could affect the glutathione uptake, which was compensated by pBAD24-gsiA. The results showed that GsiA was essential for GSI mediated glutathione uptake. GGT might mediate more glutathione uptake than GSI system.

## 4. Discussion

Glutathione is the most important antioxidant in cell and plays a plethora of cellular roles.* Salmonella enterica* can synthesize glutathione. However, there is a glutathione import like system in* Salmonella enterica*, which has never been investigated. GsiA is the ATP binding protein of glutathione importer. Putting deep insights into GsiA will help to elucidate the mechanism of glutathione import.

plou3 is derived from pMAL-c2X and used as expression vector. MBP fusion can assist protein folding and be purified specifically by MBP column. MBP used here can promote soluble expression of GsiA and make GsiA purification more convenient.

Lower IPTG concentrations and inducing temperature could contribute to the yield of soluble protein. When expressed in BL21(DE3) at 20°C with 0.1 mM IPTG, GsiA-MBP was mainly localized in soluble cellular fraction. The GsiA-MBP protein expression was confirmed by Western blot.

The Ni^2+^-NTA column was efficient at enriching the His-tagged GsiA-MBP, comprising more than 90% of total proteins. By skillfully using tags and columns, the purification of proteins could be convenient and efficient. Achievement of sufficient GsiA made future biochemical and biophysical studies possible. The ATPase activity of GsiA was measured by recording NADH oxidation. Characterization of GsiA would facilitate future determination of active sites.

To determine the in vivo function of* gisA*, the gene was deleted in* Salmonella enterica*. The growth condition of* ΔgsiA* was not affected when glutathione was used as sole sulfur source. This might be because GGT could hydrolyze glutathione to liberate glutamic acid and cysteinylglycine [25]. Cysteinylglycine was then taken up into cytoplasm and cleaved into cysteine and glycine by aminopeptidases A, B, and N and dipeptidase D, which could be utilized as a source of cysteine and glycine. However, the growth of* ΔgsiAΔggt* strain was affected when glutathione served as sole sulfur source. The glutathione uptake in* ΔgsiAΔggt* strain was undetectable. The defects could be compensated by pBAD24-gsiA. The results showed that GsiA was essential for glutathione import by GSI.

It is surprising that compared with wild type strain the CFU counts of viable bacteria of* ΔgsiA* and* ΔgsiAΔggt* in livers and spleens both undergo pronounced decrease. This might be related to the virulence of* Salmonella enterica*. Considering the functions in other pathogens [3], glutathione here was supposed to work as a signal involved in strain invasion. The pathogen might sense the host environment by importing glutathione. As* Salmonella enterica* could synthesize glutathione, it was supposed that the glutathione concentration might be used as the signal.

Collectively, an ATPase GsiA from* Salmonella enterica* was identified and characterized for the first time. Investigation of protein interaction and biological functions of GsiA might help to elucidate the glutathione import mechanism. Glutathione import involved in the virulence of* Salmonella enterica* was determined for the first time. The mechanism by which glutathione was imported and how glutathione promotes virulence of* Salmonella enterica* still need further investigation.

## Figures and Tables

**Table 1:** Primers for *gsiA* expression, deletion, and protein interactions.

Primer	Sequence 5′-3′
gsiA del-F	TGGGGATATGCCGGGGATACGCCGACCACAGGAATTTACCG GGAATAAGGAGCCGCAGGTGTAGGCTGGAGCTGCTTC
gsiA del-R	CATTTATGCGTAATAAATTGCGTCATGTTGTTCTCCTGAAACC CTGCCTGTCTGAATGCATATGAATATCCTCCTTAG
ggt del-F	CGTTGTGGGCCAGGATTAAACGGGACATACACCCATTTATCT GGAGAAAAACAACGGTGTAGGCTGGAGCTGCTTC
ggt del-R	TACTCACGGCATAATCTGCAAATAGCCCGGTGTCTGACAC- CGGGCCTGGAATTAAAAACATATGAATATCCTCCTTAG
ggt-F	ATGAAACCAACGTTTATGCGC
ggt-R	TCAGTATCCCGCCGTTAAATC
gsiA-F1	CATG CCATGG CA ATGCCGCACAGCGATGAAC
gsiA-R1	CCC AAGC TTATAAGCGCGAGAGCGCATT
gsiA-F2	CCGCTCGAG ATGCCGCACAGCGATGAAC
gsiA-R2	TCA CCCGGGTTATAAGCGCGAGAGCGCAT
gsiA-F3	CATGCCATGGCA ATGCCGCACAGCGATGAAC
gsiA-R3	CATG GACGTC TAAGCGCGAGAGCGCATTATC
gsiB-F	CATGCCATGGCA ATGACGCAATTTATTACGCATAAAT
gsiB-R	CATG GACGTC CTTCAAATCCGCATCGTCAAAG
gsiC-F1	CCGCTCGAG ATGCTTAACTATGTTCTCAAGCGC
gsiC-R1	CGCGGATCC TTACTTATACCTGATAGCCGGATTA
gsiC-F2	CATGCCATGGCA ATGCTTAACTATGTTCTCAAGCGC
gsiC-R2	CATG GCATGCCTTATACCTGATAGCCGGATTAATG
gsiD-F1	CCGCTCGAGATGCGATTATTTAACTGGCGC
gsiD-R1	CGCGGATCCTTACCCCTTAATCTTCGGGTCC
gsiD-F2	CATGCCATGGCA ATGCGATTATTTAACTGGCGC
gsiD-R2	CATG GACGTC CCCCTTAATCTTCGGGTCCAG
